# Uniportal video-assisted thoracoscopic lobectomy: An alternative surgical method for pulmonary carcinoma

**DOI:** 10.12669/pjms.325.10415

**Published:** 2016

**Authors:** Fengwu Lin, Chuan Zhang, Qiang Zhang, Kunpeng Cheng, Yan Zhao

**Affiliations:** 1 Fengwu Lin, Department of Thoracic Surgery, China-Japan Union Hospital of Jilin University, Changchun 130033, China; 2 Chuan Zhang, Department of Pediatric Surgery, The First Hospital of Jilin University, Changchun 130021, China; 3 Qiang Zhang, Department of Thoracic Surgery, The Forth Hospital of Jilin University, Changchun 130033, China; 4 Kunpeng Cheng, Department of Thoracic Surgery, The Forth Hospital of Jilin University, Changchun 130033, China; 5 Yan Zhao, Department of Endocrine, The Second Hospital of Jilin University, Changchun 130041, China

**Keywords:** Uniportal, Video assisted thoracic surgery, Lobectomy, Lung cancer

## Abstract

**Objectives:**

To explore the effects and feasibility of single-port video-assisted thoracic surgery (VATS) on lobectomy for pulmonary carcinoma.

**Methods:**

A total of 67 patients were enrolled in this study, in which 21 patients were treated by single-port VATS (Sing-port Group) and 46 patients by double-port VATS (Double-port Group). Blood loss, duration of thoracic drainage, length of post-operative hospital stay and post-operative pain ratings were compared between the two groups.

**Results:**

No significant difference existed in blood loss, duration of thoracic drainage and length of postoperative hospital stay between the two groups. However, Post-operative pain was significantly reduced in Single-port Group compared to Double-port Group.

**Conclusion:**

Single-port VATS was totally feasible with reduced post-operative pain and good looking appearance.

## INTRODUCTION

A growing number of reports have shown more advantages of video-assisted thoracic surgery (VATS) than thoracotomy on clinical indicators.^1^,^2^ And with the development of technology in VATS, operating methods of VATS are evolving from four port to uniport.^3^,^4^ Especially, uniportal video-assisted thoracoscopic lobectomy has been developed in recent years, featured by minimal invasion and difficult operation. Hereon, 67 patients who had undergone single-portal VATS (Sing-port Group) or double-portal VATS (Double-port Group) were analyzed.

## METHODS

### Patients

From October of 2013 to April of 2014, 67 patients treated with VATS were enrolled in China-Japan Union Hospital of Jilin University, in wich 21 patients were treated by single-port VATS (Sing-port Group) and 46 patients by double-port VATS (Double-port Group). All enrolled patients were free of haematological diseases, dysfunctions of heart, liver, spleen, kidney, stomach or intestine, or history of thoracic injury or operation. Each patient signed an informed consent Form. Approval was obtained from the institutional review committee of Jilin University. The patients were divided into two groups according to the surgical methods: 21 who were treated by single-port VATS (Sing-port Gruop) and 46 patients by double-port VATS (Double-port Group) (Table-I).

**Table-I:** Baseline characteristics of study patients.

*Parameters*	*Sing-port Group*	*Double-port Group*	*P value*
Age (year)	59±7.3	62±6.2	0.087
Gender(m/f)	13/8	29/17	0.929
Lobe(left/right)	11/10	19 /27	0.398

*P < 0.05 means significant difference.

### Surgical strategy and technique

All patients in the two groups were anaesthetized by the double lumen intubation method and they were placed in the maximally flexed lateral decubitus position tilted slightly backward to prevent the hip from obstructing downward movement. In Single-portal Group, a 3.0 to 5.0 centimeter incision was used as operating and camera hole. In other words, Operating instruments and camera lens were put in the same hole. Pulmonary artery was processed at first in patients with well-developed fissurae interlobaris, and then pulmonary vein and bronchus were processed. In Double Group, another incision of 1.5 centimeter was needed at the seventh interspace along mid-axillary line.

### Outcome measurements

Outcome measurements consisted of mean operation duration, length of postoperative stay, operative blood loss, duration of thoracic drainage as well as postoperative pain score in both groups. Postoperative pain questionaires were recorded by the pain grading method of the World Health Organization (WHO). Briefly, there was no pain in the 0 class. In the I class (mild pain), pain was tolerant, and it did not disturb sleeping or limit daily activity, and people could work. In the II class (middle pain), pain was obvious, and it disturbed sleeping, and people usually required general analgesic, sedative, hypnotic drugs. In the III class (severe), pain was acute with autonomic nerve functional disturbance, and it disturbed sleeping dramatically, and people usually required narcotic drugs.

### Statistics

Quantitative variables were expressed as mean±standard deviation (±s) and analyzed by t-test. Qualitative variables consisted of gender, postoperative pain score and were analyzed by χ^2^ test. A value of *P* <0.05 was considered statistically significant. All data were processed by SPSS 17.0 (SPSS Inc., Chicago, IL, USA).

## RESULTS

All cases by VATS were operated successfully without conversion to thoracotomy in both groups. And no postoperative death occurred in both groups. Postoperative pathology showed 17 lung cancers, two inflammatory pseudotumors, one pulmonary tuberculosis as well as one carcinoma metastaticum in the Uniportal Group, and 35 pulmonary carcinomas, 6 inflammatory pseudotumors, three pulmonary tuberculosis as well as two carcinoma metastaticum in the Double-portal Group.

No significant difference happened in blood loss, duration of chest drainage and length of postoperative hospital stay between the two groups. However, Post-operative pain was significantly decreased in Single-port Group compared to Double-port Group. (*P* < 0.05). Meanwhile, operation time was longer significantly in the Uniportal Group than in the Double-portal Group (*P* < 0.05) (Table-II, III).

**Table-II:** Efficacy of the two groups.

	*Sing-port Group*	*Double-port Group*	*P value*
Operation duration(min)	132.3±13.2	105.4±12.5	0.012
Blood loss (ml)	115.5±145	109.3±132	0.089
LOS* (day)	7.8±1.6	7.2±1.3	0.108
Chest tube duration (day)	4.9±1.4	4.4±1.2	0.138

* LOS: Length of postoperative stay,

P < 0.05 means significant difference.

**Table-III:** Postoperative pain ratings.

*Pain score*	*Uniportal Group*	*Double-portal Group*	*P value*
0 ~I	18	3	0.029
II ~III	29	17	

*P < 0.05 means significant difference.

## DISCUSSION

Uni-portal VATS was for the first time applied in the diagnosis of hydrothorax and pulmonary lesions. Rocco et al.^5^ reported the usage of uniportal VATS on partial lobectomy of lung, followed by thoracic sympathectomy, fenestration of pericardium and Mediastinal lymph node biopsy.^6^-^10^ Then till 2010, D Gonzalez had completed pulmonary lobectomy and even more complex operations by uniportal VATS.^11^-^14^ Based on previous developed VATS techniques,^15^ we also developed the uniportal VATS on pulmonary lobectomy and summarized up our own experience in recent years.

This study observed that postoperative pain was significantly lower in Single-port Group than Double-port Group. Previous studies^16^-^18^ have suggested postoperative pain is caused due to compression of intercostal nerve. So if hinder handle hole is removed and camera hole is integrated into frontal handle hole of 5.0 centimeters, postoperative pain would be reduced apparently, which has been confirmed in this study. On the other hand, the procedure duration was longer in the Uniportal Group than in the Double-portal Group. We suspected that it may be related to relatively long operation durations of the first few cases because of unskilled surgical techniques in uniportal VATS. Meanwhile, there existed no significant differences in intraoperative blood loss, duration of thoracic tube drainage and mean length of postoperative stay.

Conclusively, the uniportal thoracoscopic lobectomy applied in this study has proved feasible with reduced postoperative pain, and worth recommending as a safe surgical technique.
